# RhoC regulates radioresistance via crosstalk of ROCK2 with the DNA repair machinery in cervical cancer

**DOI:** 10.1186/s13046-019-1385-7

**Published:** 2019-09-05

**Authors:** Annapurna Pranatharthi, Pavana Thomas, Avinash H. Udayashankar, Chandra Bhavani, Srinag Bangalore Suresh, Sudhir Krishna, Jayashree Thatte, Nirmala Srikantia, Cecil R. Ross, Sweta Srivastava

**Affiliations:** 1 Translational and Molecular Biology Laboratory (TMBL), Department of Transfusion Medicine and Immunohematology, St. John’s Medical College Hospital (SJMCH), Bangalore, 560034 India; 2 National Centre for Biological Sciences (NCBS), Bangalore, 560065 India; 3 Rajiv Gandhi University of Health Sciences, Bangalore, 560041 India; 4 School of Integrative Health Sciences, The University of Trans-Disciplinary Health Sciences and Technology (TDU), Bangalore, 560064 India; 5 Translational and Molecular Biology Laboratory (TMBL), St. John’s Research Institute (SJRI), Bangalore, 560034 India; 6 Department of Radiation Oncology, St John’s Medical College Hospital (SJMCH), Bangalore, 560034 India; 7 Translational and Molecular Biology Laboratory (TMBL), Department of Medicine, St. John’s Medical College Hospital (SJMCH), Bangalore, 560034 India

**Keywords:** Radioresistance, DNA repair, RhoC ROCK2, Cervical cancer

## Abstract

**Background:**

Radioresistance remains a challenge to the successful treatment of various tumors. Intrinsic factors like alterations in signaling pathways regulate response to radiation. RhoC, which has been shown to modulate several tumor phenotypes has been investigated in this report for its role in radioresistance. In vitro and clinical sample-based studies have been performed to understand its contribution to radiation response in cervical cancer and this is the first report to establish the role of RhoC and its effector ROCK2 in cervical cancer radiation response.

**Methods:**

Biochemical, transcriptomic and immunological approaches including flow cytometry and immunofluorescence were used to understand the role of RhoC and ROCK2. RhoC variants, siRNA and chemical inhibitors were used to alter the function of RhoC and ROCK2. Transcriptomic profiling was performed to understand the gene expression pattern of the cells. Live sorting using an intracellular antigen has been developed to isolate the cells for transcriptomic studies.

**Results:**

Enhanced expression of RhoC conferred radioprotection on the tumor cells while inhibition of RhoC resulted in sensitization of cells to radiation. The RhoC overexpressing cells had a better DNA repair machinery as observed using transcriptomic analysis. Similarly, overexpression of ROCK2, protected tumor cells against radiation while its inhibition increased radiosensitivity in vitro. Further investigations revealed that ROCK2 inhibition abolished the radioresistance phenotype, conferred by RhoC on SiHa cells, confirming that it is a downstream effector of RhoC in this context. Additionally, transcriptional analysis of the live sorted ROCK2 high and ROCK2 low expressing SiHa cells revealed an upregulation of the DNA repair pathway proteins. Consequently, inhibition of ROCK2 resulted in reduced expression of pH2Ax and MRN complex proteins, critical to repair of double strand breaks. Clinical sample-based studies also demonstrated that ROCK2 inhibition sensitizes tumor cells to irradiation.

**Conclusions:**

Our data primarily indicates that RhoC and ROCK2 signaling is important for the radioresistance phenotype in cervical cancer tumor cells and is regulated via association of ROCK2 with the proteins of DNA repair pathway involving pH2Ax, MRE11 and RAD50 proteins, partly offering insights into the mechanism of radioresistance in tumor cells. These findings highlight RhoC-ROCK2 signaling involvement in DNA repair and urge the need for development of these molecules as targets to alleviate the non-responsiveness of cervical cancer tumor cells to irradiation treatment.

**Electronic supplementary material:**

The online version of this article (10.1186/s13046-019-1385-7) contains supplementary material, which is available to authorized users.

## Background

Cervical cancer is the most frequently diagnosed cancer in women in the underdeveloped and developing nations of the world today [1]. This disease is on the rise with approximately 85% of new cases reported in the less developed regions of the world [2]. As per the Cancer Facts & Figures 2019, American Cancer Society, the loco-regional response is very good (92%), however, the 5-year survival is 56% and 17% when diagnosed with regional and distant stage disease respectively. In India, women mostly present with a regional spread of the disease and have a poor 5 -year survival rate of 46% [3]. Concurrent chemoradiation (CCRT) is the standard of care for patients in FIGO stages IB2 to IVA. However, it must be noted that CCRT is therapeutically limited by the stage of cancer as the size of the tumor has been shown to be an important prognostic factor [4–6]. Additionally, tumor heterogeneity due to intrinsic molecular mechanisms leading to radioresistance may contribute to further CCRT limitations. This leads to the question- what can be done besides CCRT to aid better prognosis of advanced tumors. Novel approaches in terms of radiosensitizers and molecular pathway targeted therapies, alone, or in combination with platinum compounds need to be identified to address this issue.

The radioresistance phenomenon has been extensively studied in several tumors and the role of various signaling pathways has been illustrated. In the lung cancer model, AKT1 has been implicated in double-strand break repair via phosphorylation of DNA-PKc [7]. Also, the expression of a fragment of XRCC4 in breast cancer was seen to control radiation response by blocking the activity of Ligase IV of the NHEJ pathway [8]. However, there is dearth of literature to explain the regulation of radioresistance in cervical cancer. One such signaling pathway that has contributed to tumor progression in several cancers is the Rho GTPase pathway.

Rho GTPases including RhoA and RhoC, are a class of highly conserved small molecule proteins that regulate various physiological processes and are also implicated in cancer progression and metastasis [9, 10]. RhoA has been associated with poor prognosis of prostate cancer [11] and its inhibition results in a decrease in tumor phenotype in gastric cancer [12]. RhoB plays an opposing role in tumor progression mostly acting as a tumor suppressor [13]. RhoC, another member of the Rho GTPase family of proteins, is known to govern both normal cell physiology and disease progression [14–19]. Binding of GTP/GDP enables the protein to swiftly transition between “ON and OFF” states respectively, thus allowing it to efficiently control various downstream signal transduction pathways [9]. RhoC has been demonstrated to regulate migration, cell cycle progression and various transcriptional networks in cancer cells [20, 21]. It is involved in the advancement of various tumor types like breast, gastric and ovarian cancer amongst others [14, 15, 22–24]. It has been ascertained that RhoC affects response to chemotherapy in the breast cancer model [15]. Proteomic studies have revealed that RhoC is overexpressed in etoposide chemo-resistant non-small cell lung cancer [25]. Interestingly, RhoC has also been reported to regulate stemness in ovarian, breast and head and neck cancers [23, 26, 27]. Our previous report shows that RhoC via Notch1 modulates angiogenesis, migration, invasion, metastasis, anoikis resistance and tumor growth in cervical cancer, leading to the progression of the disease [28]. One of the most interesting observations was made by Hakem et al., who showed that RhoC is dispensable during the embryogenesis and initiation of a tumor but a mandate for metastasis [29]. With such diverse functions it is intuitively easy to believe that RhoC may also regulate radioresistance, however there is no report on the role of RhoC in radioresistance.

Similarly, the role of Rho-associated kinases (ROCK) - effectors of Rho GTPases, has also been investigated extensively in cancers for more than a decade now. It has been reported that targeting ROCK signaling in melanomas led to decreased growth and metastasis [30]. In bladder cancers, increased RhoA, RhoC, and ROCK signaling have been correlated with invasion and metastasis in clinical samples [31]. ROCK1 and ROCK2 have been shown to play opposing roles in glioblastomas, where the knockdown of ROCK2 was seen to enhance proliferation whereas the inhibition of ROCK1 was seen to decrease the proliferation of glioblastoma cells [32]. ROCK2 in association with CDK2, Cyclin E, NPM, and PLK2 has been shown to be important in the regulation of centrosomal duplication in varied cellular backgrounds [33–36] suggesting its active role in cell cycle regulation.

In this study, our efforts are directed towards delineating the specific role of the RhoC-ROCK2 signaling in radiation response in cervical cancer. Radiation resistance remains to be the major challenge to successful treatment of cervical cancer. Ironically, there is no biomarker to predict the outcome of radiation therapy in cervical cancer. Considering that RhoC regulates several tumor phenotypes in cervical cancer and other tumors, we decided to explore the role of RhoC as a modulator of radiation response in cervical cancer using cell lines and patient-derived cells. Our results confirm that overexpression of RhoC induces radioresistance in cervical cancer cells and ROCK2 is the downstream target of RhoC in radiation response. We demonstrate that inhibition of ROCK2 sensitizes tumor cells to radiation therapy and that the RhoC-ROCK2 signaling pathway is of crucial importance in regulation of DNA repair in cervical cancer.

## Materials and methods

### Cell lines and reagents

SiHa and CaSki (cervical squamous cell carcinoma (SCC)), cell lines used in this study were cultured using Dulbecco’s Modified Eagle Medium (DMEM) supplemented with 10% FBS (Fetal Bovine Serum) at 37 °C in 5% CO_2_ conditions. The cultures were routinely tested for mycoplasma contamination. Linear accelerator (LINAC) was used for irradiation of cell lines and patient samples. Cell viability assays were performed using WST-1 reagent (Roche). Y27632, a general inhibitor of ROCK was obtained from Calbiochem (CAS 146986–50-7). The primary antibodies used were ROCK1(sc-5560), ROCK2(sc-5561), ROCK2(CST-9029), ROCK2(sc-398519), MRE11(CST-4847), NBS1(CST-3001), RAD50(CST-3427), C-PARP (CST-9541), pH2Ax (Calbiochem DR1017), DNA-PK (CST-4602), β-ACTIN (Sigma, clone AC-74), Tubulin (Sigma N6786), RhoC (CST-D40E4), Histone 3 (CST-D1H2), CDK1 (sc-54), pCDK1(CST-9114), pP53-Ser15 (CST-9284), pAKT-Ser473 (CST-9271) and GAPDH (sc-47724). Annexin-V FITC conjugate was used from BD (556420) and Propidium Iodide (PI) from Sigma (P4170). pCAG-ROCK2 construct was a kind gift from Professor Anne Ridley, King’s College London.

### Cell survival assay

Equal number of control and experimental cells (1 × 10^3^ cells) were seeded in a 96-well format, 10 μl of WST1 was used for every 200 μl of media and incubation was done at 37 °C, 5% CO_2_ for 30 min-1 h. The plate was read using a microplate reader at 450 nm with background subtraction of 655 nm.

### Clonogenic assay

Equal numbers (1 × 10^3^ cells) of SiHa-Neo (SiHa-N) and SiHa-RhoC (SiHa-R) cells were seeded in 90 mm sterile dishes and cultured at 37 °C under 5% CO_2_ conditions for 2 weeks. In the case of irradiation preceding seeding, 35 mm dishes were used. The colonies thus formed were fixed in 4% paraformaldehyde (PFA), stained using 0.05% crystal violet, imaged and counted.

### Real time quantitative PCR

RNA isolation was performed using the TRIzol method as per the manufacturer’s protocol (Life technologies, Invitrogen). M-MLV reverse transcriptase was used for the conversion to cDNA according to the manufacturer’s protocol (Life technologies, Invitrogen). Gene expression was studied by qPCR using the Power SYBR green fast master mix and run on 7500 Fast Real Time PCR by Applied Biosystems. Primer sequences used in the experiments are tabulated in Table 1.

**Table 1:** Sequences of primers used

Primer Name	Sequence-5’-3’
ROCK2 Forward primer	GAGAGCTTGCTGGATGGCTT
ROCK2 Reverse primer	CGAACCAACTGCACTTCACC
BRCA2 Forward primer	GAAGCGTGAGGGGACAGATT
BRCA2 Reverse primer	ATCTGCTTTGTTGCAGCGTG
RAD50 Forward primer	AAACTGCGACTTGCTCCAGA
RAD50 Reverse primer	TGTCGTTCTTTAGGCGCTGT
NBS1 Forward primer	ACCAACCTGAGTCAAACAGATGA
NBS1 Reverse primer	GAGCATGCAACCAAAGGCTC
GAPDH Forward primer	GAAGGTGAAGGTCGGAGTC
GAPDH Reverse primer	GAAGATGGTGATGGGATTTC
RPLP Forward primer	GCGACCTGGAAGTCCAACT
RPLP Reverse primer	CCATCAGCACCACAGCCTTCC
ROCK2 Forward primer (mouse)	GAGTCTGCTGGATGGCTTAAA
ROCK2 Reverse primer (mouse)	TTCACCAAAAGCACCTCTTCCA

### Immunoblotting

Cells washed with cold 1xPBS were incubated with lysis buffer (20 mM Tris–HCl (pH 7.5), 150 mM NaCl, 1%(v/v) NP-40, 1%(w/v) sodium deoxycholate, 0.1% (w/v) SDS, 50 mM NaF, 1mM Na_3_VO_4_, 50 mg/ml PMSF, 1 mg/ml leupeptin, 1 mg/ml pepstatin) for 30 min on ice, homogenized with a 23-G needle and centrifuged at 14,000 rpm for 10 min at 4 °C. The lysate was resolved using SDS–PAGE, blotted and probed using the appropriate antibody. Fractionation westerns were performed as described in Suzuki et al., [37] Histone3 and alpha-Tubulin were used as loading controls for the nuclear fraction and cytoplasmic fractions respectively.

### Pre-extraction of cells

Prextraction of cells was performed after washing cells twice with 1xPBS. The cells were treated with CSK buffer [38] twice for 5 min each and then washed with 1xPBS. The cells were then fixed in 2% PFA for further use.

### Immunofluorescence

Cells were fixed in 4% PFA and washed with 1xPBS prior to staining. The cells were permeabilized using 0.2% Triton-X in 1xPBS (PBST) for 5 min. The cells were blocked using 10% FBS for 1 h. Primary antibodies were added and incubated at RT for 1-2 h. The cells were washed with 1xPBST. Secondary fluorescent antibodies were used at 1:500 dilution and incubated at RT for 45 min. The cells were washed with 1xPBS and mounted using anti-fade gold (Thermofisher Scientific).

Immunofluorescence analysis was performed on cryo-sections as described previously [39] from patient samples using citrate buffer (pH 6.0) for antigen retrieval by boiling for 20 min. The slides were cooled at RT for 30 min prior to permeabilization using 0.2% Triton-X 100 in 1xPBS. Following this, blocking was done in 10% FBS and primary antibodies were used at the requisite dilutions. The slides were then incubated overnight at 4 °C. After washes in 1xPBS, the secondary staining was done using secondary Alexa fluorophores and vectashield was used for mounting. Images were taken using the Zeiss 710 confocal microscope.

### Antibody inhibition assay

Antibody inhibition using saponin has been performed for the first time to the best of our knowledge. The cells were live permeabilized using 1 μg of the ROCK2 antibody, resuspended in 1xDMEM complete media (10%FBS) containing 0.0025% saponin. The media was replaced with 1xDMEM complete media devoid of saponin after 18 h. IgG was used as the isotype control. The reduction in protein levels of ROCK2 was confirmed by immunoblotting.

The specific antibody binding was confirmed by permeabilizing the cells either with the isotype or the ROCK2 antibodies using 0.0025% saponin in 1xPBS for 1 h, following which the cells were lysed and incubated with 1.5 mg of Dyna beads for overnight at 4 °C. The eluate from the Dyna beads was then immunoblotted and probed using ROCK2 antibody.

#### Clinical specimens

The primary tumor samples obtained from patients were subjected to collagenase treatment at a concentration of 0.2 mg/ml using a magnetic stirrer at 37 °C until single cells were obtained. The cells were passed through cell filtration unit and the single cells thus obtained were counted. The cells were lineage depleted using the manufacturer’s protocol (Miltenyi biotech-lineage depletion kit human). The cells were then inhibited using the ROCK2 antibody followed by 6Gy irradiation on the next day and the cell survival was assayed after 3 days.

### Flow cytometry

For flow cytometric analysis cells were stained using the immunofluorescence protocol as described above. The cells were acquired using Gallios or FC500 flow cytometers. Cell cycle analysis was performed using DRAQ5/Hoechst33342 according to the manufacturer’s protocol. The cell viability tests were performed using either Annexin V, Propidium Iodide (PI-1 mg/ml) or both.

### Live cell sorting

We developed a novel method to sort live cells based on the intracellular antigen ROCK2 followed by culturing for further assays. For live cell staining of the intracellular antigen prior to sorting, cells were detached using 5 mM EDTA, counted and 2 × 10^6^ cells were used per tube. The cells were then spun at 1500 rpm for 5 min at 4 °C and 0.0025% saponin in 1xPBS was added to the cells for 10 min. The cells were centrifuged at 1500 rpm for 5 min at 4 °C. The supernatant was completely drained. The saponin solution containing 1 μg of the antibody was added to the cells and incubated at RT for 1 h. Following incubation, the cells were washed with 1xPBS twice and the secondary antibody was added at 1:500 dilution for 30 min. After three washes with 1xPBS, the cells were taken for sorting in 2% serum containing 1xPBS. Live sorting was performed using BD Aria flow cytometer.

### Migration assay

Migration assay was performed in a 6 well transwell chamber (8-μm pore size) using 1 x10^5^cells that were seeded on the top chamber in 1% FBS containing 1xDMEM. The bottom chamber contained 10% FBS in 1xDMEM. The cells were incubated for 16 h in the incubator at 37 °C under 5% CO2 conditions. At the specified time point the cells were fixed using 4% PFA followed by DAPI staining of the cells that migrated across the membrane, towards 10% FBS containing media. DAPI stained cells that migrated were counted for the estimation of number of migratory cells.

### Transcriptomic and bioinformatic analysis

The transcriptomic analysis was performed using Illumina paired end sequencing (150 × 2). The sequenced reads were aligned to the *Homo sapiens* DRCh38 build genome downloaded from Ensemble database. An average of 91.77% of the reads aligned to the reference genome. Tophat was used to align the transcript sequences and cufflinks were used to create a combined assembly. A Differential Gene Expression (DGE) analysis was performed using Cuffdiff package. Using DAVID, a gene ontology analysis was performed for the upregulated genes and the genes that were specifically expressed in the treated pool. Heatmap analysis was done for the DGE genes, using Clustvis, R based bioinformatic tool. The transcriptomic analysis was performed in replicates of *n* = 2. STRING database (version 11.0) was used to study the interaction networks.

### Xenograft assays

2 × 10^6^ cells of both irradiated (IR) and non-irradiated (NR) SiHa cells were embedded in Matrigel to grow tumors subcutaneously in SCID mice. After 4 weeks mice were sacrificed, tumors excised and weighed. The tumors were fixed using PFA, cryo-sectioned and stained using routine immunofluorescence procedures as described earlier for the patient sample sections. Imaging was done using Zeiss 710 confocal microscope.

### Statistical analysis

The mean and standard deviations have been computed for the experiments performed in triplicates and the significance was calculated using the t-test. *p* < 0.05 was considered significant.

## Results

### RhoC governs the transcriptional network in cervical cancer cell line

Heterogeneous response to concurrent chemoradiation therapy (CCRT) is governed by the tumor stage and molecular heterogeneity within the tumor, consequently leading to poor prognosis in cervical cancer. The challenge to successful treatment of this disease is dependent on identifying signaling pathway alterations which regulate the resistance phenotype. We have earlier published that RhoC regulates tumor progression in cervical cancer [28]. In the present study, we explore the role of RhoC as a regulator of radioresistance.

**Fig. 1:**
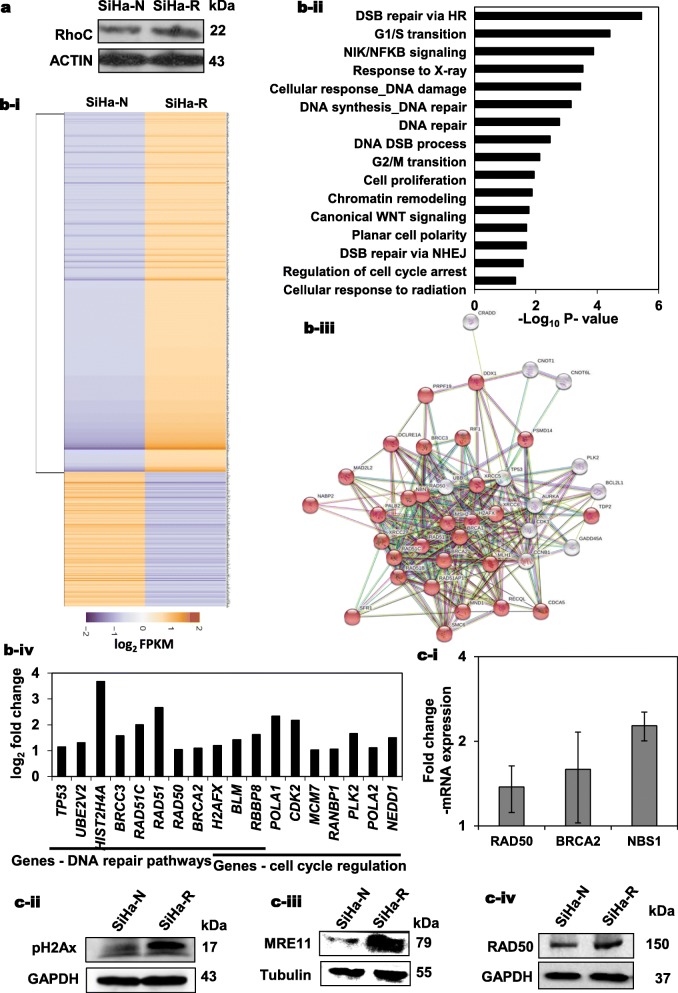
Transcriptional enrichment of DNA repair genes upon RhoC overexpression. **a** Immunoblot analysis of the SiHa-R cells shows increased RhoC as compared to SiHa-N cells which confirms stable over-expression of RhoC (*n* = 3). **b-i** A heatmap representation of gene expression patterns using Log_2_ FPKM of the SiHa-R versus SiHa-N cells transcriptomic data, using Clustvis analysis tool. **b-ii** Genes upregulated in SiHa-R cells were subjected to GO analysis using DAVID and only the clusters with a P significance (*p* < 0.05) have been represented. GO terms for the biological processes with their corresponding -Log10 P- value scores have been depicted in the graph. **b-iii** String interaction analysis was performed using version 11.0. DNA repair and cell cycle pathways were enriched with a *p* < 0.05 using DAVID annotation tool. The enriched genes were used for the string analysis where red nodes are suggestive of the tight clustering in the network. The confidence level was set to 0.4 (medium). **b-iv** Graphical representation of a few selected genes from the battery of genes involved in DNA repair and cell cycle regulation which were significantly upregulated in SiHa-R cells (log_2_ fold change> 1, *p* < 0.05). **c-i** Real-time PCR based validation of some of the representative genes upregulated in SiHa-R cells. SiHa-N was used to normalize the expression levels. **c (ii-iv)** Cellular extracts of SiHa-R and SiHa-N cells were analyzed for DNA repair proteins. The expression levels of pH2Ax, MRE11 and RAD50 in SiHa-R cells were higher as compared to the control cells (*n* = 3)

Cell lines over-expressing the RhoC gene and its variants [28], were used to understand the role of RhoC in radioresistance. Transcriptional analysis was performed on SiHa cells, either overexpressing RhoC or harbouring only pCDNA3.0. Western blot analysis confirmed that SiHa-R cells have increased levels of the RhoC protein (Fig. 1a). As shown in Fig. 1b-i, Clustvis enabled heatmap analysis [40] of the differentially expressed genes (DEGs) with threshold fold change > 1.5 and < 0.5 shows a distinct gene expression pattern between the cell lines. 1627 genes (*p* < 0.05) were upregulated and 424 genes (*p* < 0.05) were down-regulated in SiHa-R cells as compared to SiHa-N cells. The number of genes upregulated was more than those that were downregulated, suggesting that RhoC positively regulates transcriptional network. Subsequently, Gene Ontology (GO) analysis using the DAVID functional annotation tool [41], was performed to understand enrichment of genes regulated by RhoC and the important biological processes that they regulate. The analysis demonstrated that genes regulated by RhoC associated with 250 biological processes including DSB repair via HR/NHEJ, G1/S transition, NIK/NFKB signaling, response to X-ray, cellular response to DNA damage and DNA repair (Fig. 1b-ii), supporting a role for RhoC in radiation induced DNA repair.

An understanding of the cellular functions requires an in depth understanding of the functional interactions between the proteins. This can be achieved by the STRING network analysis**.** Thus, the enriched genes were further uploaded on STRING [42] for visualization of the interaction network that may be driven by RhoC. Selection of DNA repair pathway (Fig. 1b-iii) highlighted the corresponding protein nodes in the network, which indicate the functional connections detected in this gene set. The genes highlighted include the important DNA repair and cell cycle genes like CDK1, TP53 and RAD50. This indicated that the RhoC overexpression regulates the DNA repair machinery in the cervical cancer cells. The selected DNA repair genes as shown in Fig. 1(b-iv) are highly upregulated in the SiHa-R cells**.** A quantitative PCR based expression analysis of a select few genes including RAD50, BRCA2 and NBS1 confirmed that these genes were indeed significantly upregulated in SiHa-R cells as compared to SiHa-N (Fig. 1c-i). Additionally, immunoblot analysis of pH2Ax, MRE11 and RAD50 also illustrated an upregulation of these proteins in SiHa-R cells (Figs. 1c(ii-iv)). pH2Ax, MRE11-RAD50-NBS1 (MRN) complex are well known regulators of DNA repair and are involved in the sensing and repairing of double-strand breaks (DSBs) in DNA [43–45]. Combined, these findings begin to define the role of RhoC in DNA repair and radiation response in cervical cancer.

### RhoC regulates radiation response in cervical cancer cells

**Fig. 2:**
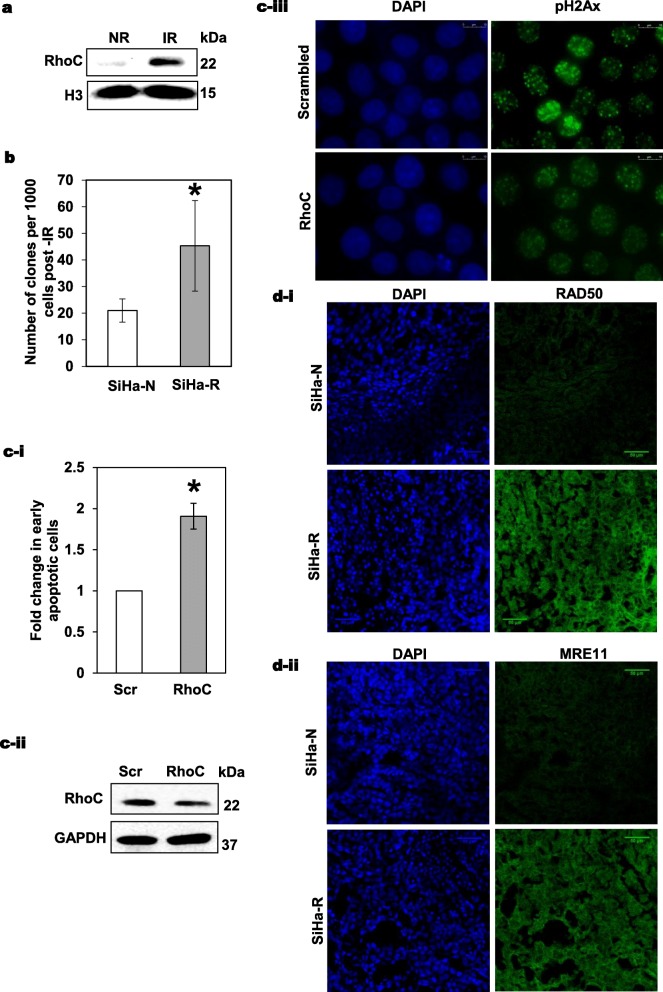
Evaluation of the effect of RhoC on radiation response in cervical cancer cells. **a** Immunoblot analysis for expression of RhoC in cellular extracts of irradiated and non-irradiated SiHa cells showed that the irradiated cells have increased RhoC expression (*n* = 3). **b** Increased colony formation by 2.1-fold is shown in irradiated SiHa-R cells as compared to cells containing the control empty vector *p < 0.05. (*n* = 3). **c-i** Graphical representation of fold change in apoptotic cells upon RhoC knockdown followed by irradiation as analyzed by flow cytometry using Annexin V. Scr-Scrambled siRNA and RhoC -RhoC siRNA. (*n* = 3, *p < 0.005). **c-ii** Immunoblot analysis shows reduction in RhoC expression levels upon siRNA- based inhibition (*n* = 3). **c-iii** SiHa cells were irradiated 72 h post transfection with RhoC and scrambled siRNA and assessed for pH2Ax foci. Immunofluorescence analysis shows a decreased pH2Ax foci formation in cells with RhoC siRNA knockdown (scale bar = 10 μm). **d(i-ii)** Immunofluorescence analysis of SiHa-N and SiHa-R xenograft sections showed an increased expression of RAD50 and MRE11 proteins (scale bar = 50 μm)

Next, we demonstrated that indeed increased RhoC overexpression modulates the radioresistance of cervical cancer cell lines. Clonogenic assay to test the cell survival and colony formation ability after irradiation showed that SiHa-R cells had better clonogenic ability as compared to SiHa-N cells (Fig. 2b), alternatively, RhoC inactivation abrogated this effect. The CaSki-dnR cells (CaSki cells containing the dominant negative form of the RhoC) showed greater sensitivity to irradiation than CaSki-N cells, harbouring backbone vector alone (Additional file 1: Figure S1a).

All irradiation experiments were carried out at 6Gy following a dose curve analysis which indicated that 6Gy was the LD50 for irradiation (Additional file 1: Figures S1b(i-ii)). The IR cells showed pH2Ax foci formation suggestive of radiation response in these cells (Additional file 1: Figure S1c). The resistance of the surviving fraction was confirmed by re-irradiating these cells followed by cell death analysis which suggested that there was no significant increase in cell death (Additional file 1: Figure S1d(i-ii)). The cells in the surviving fraction also showed enhanced clonogenic ability (Additional file 1: Figure S1e-i), and increased migration (Additional file 1: Figure S1e-ii). Considering that RhoC expression modulated radiation resistance of the tumor cells, we examined the changes in RhoC expression in the irradiated cells. We found an increased expression of this molecule as shown in Fig. 2a and Additional file 1: Figure S2a(i-ii).

To further demonstrate the contribution of RhoC to radiation response in cervical carcinoma, we used RhoC siRNA-based knockdown to evaluate its effect on cell survival (Fig. 2c-i). The SiHa cells treated with RhoC siRNA showed an increased apoptosis following irradiation thereby confirming that RhoC regulates radiation response in these cells. The specificity of the RhoC siRNA (previously published [28];) was re-confirmed by immunodetection of both RhoC and RhoA (Fig. 2c-ii and Additional file 1: Figure S2b(i-ii)). Additionally, the knockdown of RhoC resulted in decreased pH2Ax foci formation in SiHa cells post-irradiation (Fig. 2c-iii). As shown in Fig. 1c-ii, the SiHa-R cells had increased pH2Ax as compared to SiHa-N cells.

We also analyzed the expression of DNA repair proteins using SiHa-R and SiHa-N xenografts, the tumor forming properties of which has been previously reported [28]. Upon immunofluorescence staining, it was observed that RAD50, MRE11 and pH2Ax proteins were highly expressed in SiHa-R as compared to SiHa-N xenografts (Figs. 2d(i-ii) and Additional file 1: Figure S2c). Alternatively, RAD50 was seen to be down-regulated in CaSki-dnR cells as compared to the CaSki-N cells (Additional file 1: Figure S2d)**.** Though the mechanism of regulation of gene expression by RhoC is not yet known, the above data reinforces our hypothesis that RhoC over-expression confers resistance to radiation by regulating the DNA repair protein expression. Combined, these findings begin to define the cellular consequences of RhoC driven transcriptional regulation of radioresistance.

### ROCK2 regulates radioresistance in cervical cancer cells

Once the radioresistant phenotype of SiHa-R cells was confirmed by both molecular and functional approaches, we attempted to identify the downstream effector of RhoC in this context. The Rho-associated kinases, ROCK1 and ROCK2, are well established downstream targets of small GTPases including RhoC [46]. These serine/threonine kinases are involved in several cellular mechanisms, with both ROCK1 and ROCK2 involved in regulation of cell migration [47], whereas ROCK2 plays a role in regulation of centromere duplication [48] and cell cycle progression via Cdc25A [49].

**Fig. 3:**
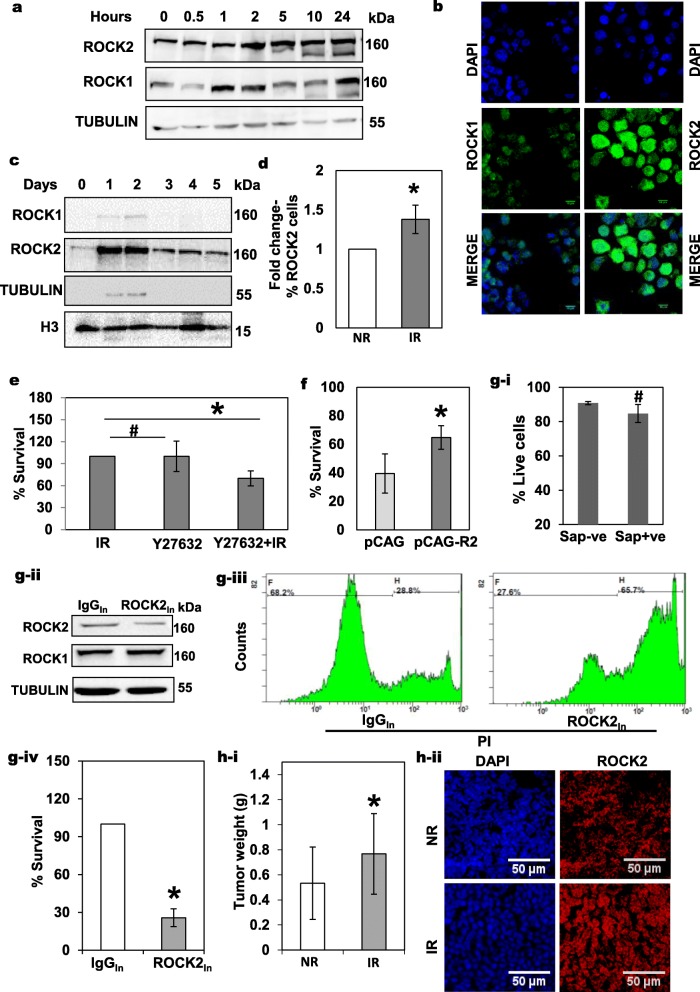
Evaluation of the role of ROCK2 in radiation response in SiHa cells. **a** Immunoblot analysis of whole cell extracts of SiHa cells showed an upregulation of both ROCK1 and ROCK2 proteins upon irradiation at the indicated time points. **b** Immunofluorescence analysis also showed that the ROCK2 expression levels were higher in the nuclear compartment as compared to the ROCK1 levels. Scale bar = 10 μm.(*n* = 3). **c** Immunoblot analysis of ROCK1 and ROCK2 at different times points in the nuclear extracts of IR SiHa cells (*n* = 3). **d** Graphical representation of flow cytometry analysis showing an increased percentage of cells with ROCK2 expression in the irradiated (IR) SiHa cells as compared to the non-irradiated (NR) control cells. A fold increase of 1.4 is depicted (*p* < 0.03; *n* = 3). **e** Inhibition of ROCK signaling using Y27632 (10 μM) to determine its effect on cell survival. There was a 30% increase in cell death of the treated cells due to irradiation (*n* = 3, *p < 0.037; # n.s). **f** SiHa cells transfected with 1 μg of pCAG-ROCK2 expression vector and the corresponding empty vector followed by irradiation displayed increased survival. (*p* < 0.01, *n* = 4). **g-i** SiHa cells treated with saponin (Sap+ve) had comparable cell viability as compared to the untreated control (Sap -ve), #- n.s (*n* = 3). **g-ii** Immunoblot analysis showed that there is reduction in ROCK2 levels but not ROCK1 upon inhibition with the ROCK2 antibody (ROCK2_In_). IgG isotype has been used as control (*n* = 3). **g-iii** Representative histograms depict the increased PI uptake in cells with ROCK2_In_ as compared to the corresponding isotype control. **g-iv** Graphical representation of flow cytometry-based analysis of cell survival upon ROCK2_In_ followed by irradiation shows increased sensitization of SiHa cells to irradiation (*n* = 3, *p* < 0.001). **h-i** Graphical representation of weights of tumors formed by NR and IR cells (NR tumor = 0.53 g ±0.28; IR tumor = 0.76 g ±0.32, *n* = 3, *p* < 0.02). **h-ii** Representative images of immunofluorescence-based analysis of xenografts derived from NR and IR tumors showed increased expression of ROCK2 in IR tumor derived sections (scale bar = 50 μm)

To identify the ROCK protein involved in DNA repair, an expression analysis of both ROCK2 and ROCK1 was performed at various time points post-irradiation. It was seen that the expression of both ROCK1 and ROCK2 increased upon irradiation, with ROCK2 being stable from 2 h up until 24 h (Fig. 3a). Immunofluorescence analysis of ROCK1 and ROCK2 in irradiated SiHa cells showed that ROCK2 was nuclear as compared to ROCK1 (Fig. 3b). Since, DNA repair is a nuclear event, a protein contributing to DNA repair would presumably have a nuclear localization. As expected, the analysis of the nuclear protein fractions of irradiated cells by biochemical fractionation, showed a marked increase in the levels of ROCK2 on Days 1 and 2 after irradiation (Fig. 3c and Additional file 1: Figure S3b). Similar results were also observed following a pre-extraction protocol for detection of nuclear ROCK2 (Additional file 1: Figure S3c). Further flow cytometric analysis of irradiated cells as shown in Fig. 3d showed an increased percentage of ROCK2 positive cells. Additionally, as expected we observed increased ROCK2 and RhoC levels in re-irradiated SiHa cells as compared to IR cells (Additional file 1: Figure S3a).

In order to study the effect of ROCK signaling on radiation response and radioresistance, we treated SiHa cells with Y27632 (ROCK inhibitor) [50]. Treatment of SiHa cells with 10 μM Y27632 resulted in sensitization of these cells to radiation, resulting in increased cell death as seen by flow cytometric analysis (Fig. 3e). To specifically implicate ROCK2 in radioresistance, experiments were undertaken to alter the expression of ROCK2 alone and analyze the effect on cell survival post-irradiation. We noted that overexpression of ROCK2 in SiHa cells conferred resistance to these cells resulting in better survival post-irradiation, whereas inhibition of this protein sensitized cells to radiation, consequently leading to increased cell death. Transfection of pCAG-ROCK2 [51] into SiHa cells, followed by irradiation resulted in decreased in cell death, indicating better resistance to irradiation in the background of ROCK2 over-expression (Fig. 3f). Q-PCR based analysis confirmed that transfection of pCAG-ROCK2 resulted in increased ROCK2 expression in SiHa cells (Additional file 1: Figure S3d). Alternatively**,** inhibition of ROCK2 via siRNA mediated silencing showed an increase in early apoptotic cells post-irradiation in comparison with the scrambled siRNA control (Additional file 1: Figure S3e(i-ii)).

To further confirm that ROCK2 is indeed important and regulates radioresistance in cervical cancer, we decided to use another approach to inhibit ROCK2 and observe the effect on cell survival. Given that Y27632 is an inhibitor of both ROCK2 and ROCK1 and siRNA has limitation with transfection of primary cells, we chose to use antibody mediated inhibition to this effect.

The use of antibodies to inhibit surface protein functions has been well explored and is currently in clinical use as targeted therapeutics [52]. As early as 1994, the technique of inhibition of intracellular cyclin D was performed in cells by microinjection of the specific antibody [53]. Also, the use of other detergents like TRITON-X have been employed for the delivery of macromolecules into the live cells [54]. We worked on similar principles and used Saponin, which is a glycoside with mild detergent properties to perform reversible permeabilization of cells. Saponin at a concentration of 0.0025% (w/v) was used for the subsequent antibody mediated inhibition to study the effect of ROCK2 inhibition on cell survival. Flow cytometric analysis of cell death proved that saponin treatment alone did not affect cell viability under both non-irradiated and irradiated conditions (Figs. 3g-i and Additional file 1: Figure S3f). Cells were then treated with the ROCK2 and isotype IgG antibody in the presence of saponin. Western blot analysis shows the specific inhibition of ROCK2 by the antibody with no effect on the ROCK1 levels (Fig. 3g-ii). Additionally, in order to establish that the antibody did indeed permeate the cells treated cells were lysed after 1 h of treatment and dynabeads were used to immunoprecipitate ROCK2. Immunoblot analysis of the immunoprecipitate confirmed that the ROCK2 protein was pulled down in ROCK2 treated cells only, with no corresponding band in the IgG-treated lane, thus confirming that the antibodies entered and bound to the ROCK2 protein within live cells (Additional file 1: Figure S3 g). To test the effect of ROCK2 inhibition on radiation response, the treated SiHa cells were irradiated and cell death analysis was performed. Significant cell death was observed in the ROCK2 inhibited (ROCK2_In_) cells as compared to the IgG control (IgG_In_), indicating sensitization of these cells to irradiation following ROCK2 antibody treatment (Fig. 3g (iii-iv)).

Additionally, we further tested the expression of ROCK2 in xenografts formed using non-irradiated (NR) versus irradiated (IR) SiHa cells. We found that the xenografts obtained from the surviving fraction of irradiated SiHa cells were significantly heavier (Figs. 3h-i and Additional file 1: Figure S3 h-i). An expression analysis using immunofluorescence and immunoblotting respectively, in sections and xenograft lysates, showed a marked increase in ROCK2 expression in the IR xenografts (Fig. 3h-ii and Additional file 1: Figure S3 h-ii). These data collectively suggest a role of ROCK2 in radiation response in cervical cancer.

### Transcriptional gene networks support DNA repair in ROCK2 high cells

DNA repair and radioresistance in tumors are closely associated [55] and therefore, we tested the association of ROCK2 with DNA repair. To understand this relationship, we used transcriptomics-based approach to understand the status of DNA repair machinery in the ROCK2 expressing cells. A transcriptomic analysis was performed on the cells sorted based on ROCK2 expression, as high ROCK2 (ROCK2_hi_) and low ROCK2 populations (ROCK2_lo_).

**Fig. 4:**
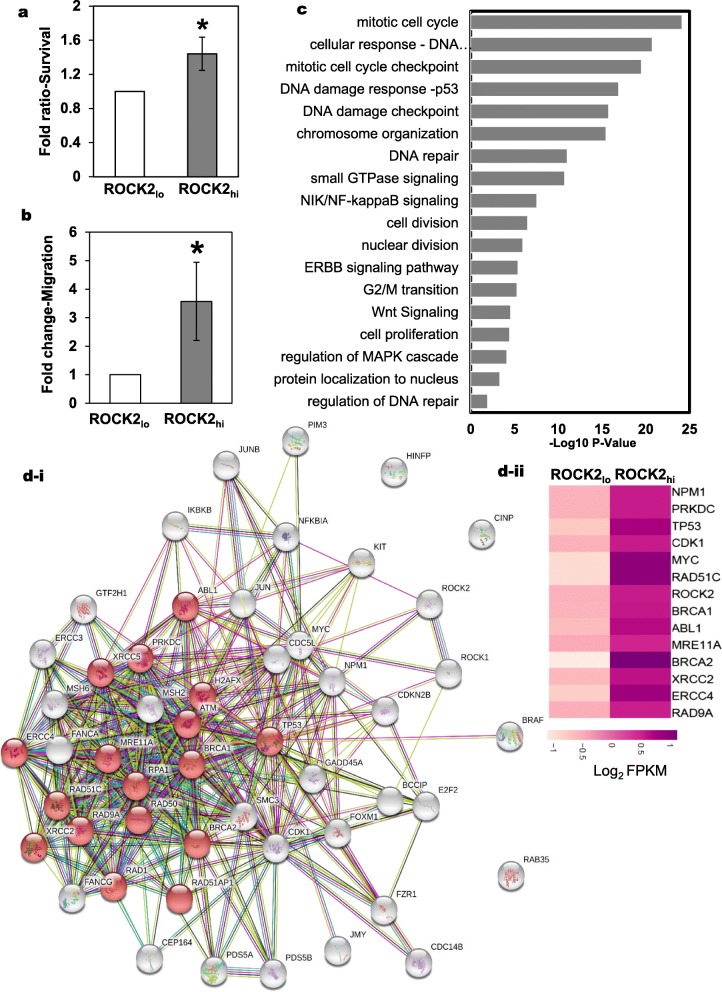
Transcriptional analysis highlights enrichment of DNA repair and survival pathway genes in ROCK2_hi_ cells. **a** A graphical representation of survival following irradiation in ROCK2_**hi**_ cells and ROCK2_**lo**_ cells. The viability was determined using the WST1 assay reagent. ROCK2_**hi**_ cells showed better survival. Fold ratio survival represented is normalized to that of the ROCK2_lo_ cells (*n* = 4, 1.4-fold, *p* < 0.03). **b** A graphical representation of increased migration of ROCK2_**hi**_ cells as compared to ROCK2_**lo**_ cells sorted based on ROCK2. *p < 0.03 (*n* = 3). **c** GO enrichment analysis of the selected biological processes in the ROCK2_hi_ cells with a *p* < 0.05 represented as a graph with -Log_10_ *P*-values plotted on x-axis. **d-i** String interactome analysis was performed on the set of 51 genes that broadly represented DNA repair, cell cycle apoptosis and cell division. The network of DNA repair proteins formed a tight cluster represented in red colored nodes. The confidence level was set to 0.4 (medium). PPI enrichment *p*-value:< 1.0e-16. **d-ii** Heatmap of the representative genes that have been used in String analysis confirmed to be upregulated in the ROCK2_hi_ cells. Clustvis webtool based on R-software was used for the analysis. Color key shows the differential expression of the genes

Live sorting of cells, based on extracellular proteins has been practiced for long, however sorting of cells using intracellular protein is a challenge. The need of this study was to sort cells based on the ROCK2 expression in order to perform transcriptomics. Since we had already developed an assay to live permeabilize antibody for the ROCK2 inhibition assay, we used the same protocol to live sort ROCK2_hi_ cells and ROCK2_lo_ cells. Live permeabilization and ROCK2 staining was performed using saponin as described in the methodology section. The gating strategy to specifically select the ROCK2_hi_ versus ROCK2_lo_ expressing SiHa cells is shown in Additional file 1: Figure S4a. Further, post sorting, these cells were imaged to confirm the differential expression of ROCK2. As shown in Additional file 1: Figure S4b, ROCK2_hi_ cells undeniably expressed higher levels of ROCK2 as compared to ROCK2_lo_ cells. This was further confirmed by Q-PCR (Additional file 1: Figure S4c-i). These cells were also examined for RhoC levels by a real time PCR and the RhoC levels were found to be enhanced as expected (Additional file 1: Figure S4c-ii). Phenotypic viability of these cells was confirmed by re-culturing and re-irradiation of these cells. Strikingly, the ROCK2_hi_ cells elicited a better cell survival advantage (Fig. 4a) whereas the ROCK2_lo_ cells comparatively exhibited increased cell death upon re-irradiation. ROCK2 is also reported to regulate migration phenotype [56], thus these cells were also tested for their migration ability. As shown in Fig. 4b, there was increased migration of ROCK2_hi_ cells as compared to ROCK2_lo_ cells when seeded in a Boyden transwell chamber.

The transcriptomic analysis of these two populations, ROCK2_lo_ and ROCK2_hi_ reflected global changes in the gene expression profiles. As shown in Additional file 1: Figure S4d, there was a distinct transcriptional network operational in ROCK2_hi_ cells as compared to ROCK2_lo_ cells. A GO annotation revealed that the differentially expressed genes clustered in several biological processes and significant amongst them were the genes involved in cellular response to DNA damage, mitotic cell cycle checkpoint, DNA damage checkpoint, DNA repair, DNA damage response-p53 and several others including WNT signaling and MAPK signaling pathways (Fig. 4c). The GO analysis provided an insight into the transcriptional status of the ROCK2_hi_ cells and indicated that these cells have an enriched DNA repair transcriptional network. STRING network analysis was used to identify the transcriptional networks in these cells. String database allows building of networks and understanding the cellular functions using information about the interaction between expressed proteins. As shown in Fig. 4d-i, the genes enriched in the GO biological processes formed a well-defined network, with some of the DNA repair pathway genes forming a tightly clustered network. This analysis confirms the transcriptional status of the ROCK2_hi_ cells and suggests a better DNA repair activity by these cells. The network also mapped the genes that were involved in cell cycle regulation and the TP53 signaling which is important in mediating a cell cycle response to DNA damage stimulus. A heatmap representation of the genes that formed a tight cluster, as shown by STRING network analysis depicts the increased expression levels of those genes in the ROCK2_hi_ population (Fig. 4d-ii). These data support the existence of better DNA repair machinery for the regulation of resistance in the cervical cancer tumor cells with higher ROCK2 expression.

### ROCK2 crosstalk with DNA repair assembly proteins to regulate radiation response in cervical cancer cells

**Fig. 5:**
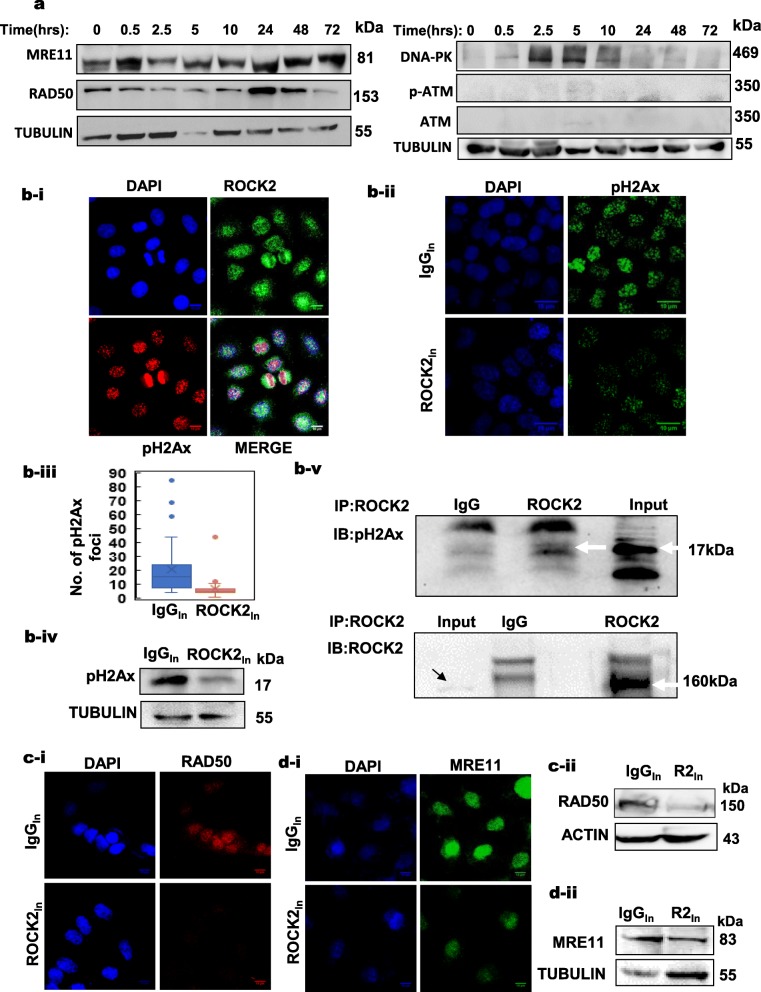
ROCK2’s crosstalk with DNA repair machinery in cervical cancer cells. **a** Immunoblot of irradiated SiHa cells showing expression levels of DNA repair proteins at various time points indicated, as compared to control at 0 h. **b-i** Representative immunofluorescence images depict the co-expression of ROCK2 and pH2Ax in CaSki cells at 1 h following irradiation (*n* = 3, scale bar = 10 μm). **b-ii** Representative immunofluorescent images of irradiated CaSki cells showing a decreased expression of pH2Ax in ROCK2_In_ as compared to IgG_In_ cells (*n* = 3, scale bar = 10 μm). **b-iii** Box plots of the number of pH2Ax foci in IgG_In_ and ROCK2_In_ cells following irradiation. A median value of 15 foci/cell in IgG_In_ as opposed to 5 foci/cell in the ROCK2_In_ was observed (*n* = 3, *p < 0.01). **b-iv** Immunoblot analysis demonstrated a reduction in pH2Ax levels at 1 h in irradiated ROCK2_In_ as compared to IgG_In_ cells (*n* = 3). **b-v** Immunoprecipitation using ROCK2 antibody shows pull down of pH2Ax at 1 h following irradiation treatment. IgG isotype is used as the control (*n* = 3). **c-i** Representative immunofluorescence images showing a decreased expression of RAD50 as compared to IgG_In_, in irradiated CaSki cells, with ROCK2_In_ (*n* = 3, scale bar = 10 μm). **c-ii** Immunoblot analysis in irradiated SiHa cells confirming reduction in RAD50 levels upon ROCK2_In_ (*n* = 3). **d-i** Representative immunofluorescence images showing a decreased expression of MRE11 as compared to IgG_In_ cells, in irradiated CaSki cells, with ROCK2_In_ (*n* = 3, scale bar = 10 μm). **d-ii** Immunoblot analysis of irradiated SiHa cells also showed a reduction in MRE11 levels upon ROCK2_In_ (*n* = 3)

Our data so far, convincingly suggests that ROCK2_hi_ cells have an enhanced DNA repair and this was further confirmed using biochemical approaches. The initial events in DNA repair comprise sensing of DNA damage followed by activation of p53 which results in cell cycle arrest at G1/S and G2/M, allowing cells to repair the DNA [57, 58]. These processes require orchestration of a large network of proteins including ATM, BRCA2/1, CHEK1/2, p53 and the RAD family proteins [59]. Gamma phosphorylation of H2Ax is one of the early events in double stand break repair pathways and is essential for the recognition and repair of DNA double strand breaks [60, 61]. We thus decided to investigate the association between the DNA repair process and ROCK2 expression. Western blot and immunofluorescence analysis revealed an upregulation of some of the important DNA repair proteins including MRE11, NBS1, RAD50 and DNA-PKc in irradiated SiHa cells, however, no distinct changes were observed in ATM levels (Fig. 5a and Additional file 1: Figure S5a). We also found that ROCK2 and pH2Ax, which is a DNA repair sensor protein, were co-expressed in irradiated cells (Fig. 5b-i). Further, ROCK2 was inhibited and its effect on the pH2Ax levels was investigated. As expected, ROCK2 inhibition resulted in decreased pH2Ax foci formation upon irradiation (Fig. 5b(ii-iii)). This was also confirmed by western blot analysis of irradiated cells, which showed a significant decrease in pH2Ax levels (Fig. 5b-iv) upon ROCK2 inhibition. Interestingly, immunoprecipitation of pH2Ax using ROCK2 antibody in the irradiated cells, confirms an interaction between them (Fig. 5b-v and Additional file 1: Figure S5b). We then tested if ROCK2 inhibition would result in downregulation of other components of the DNA repair machinery. The MRN complex, comprising of MRE11, RAD50 and NBS1, is a highly conserved protein complex that plays a major role in sensing and processing DSBs [62]. Immunoblot and immunofluorescence analysis of MRE11 and RAD50 in irradiated cells revealed a significant reduction in the levels of these proteins upon ROCK2 inhibition. The levels of RAD50 protein were seen to reduce considerably upon ROCK2 inhibition (Fig. 5c-i). Western blotting further confirmed this result (Fig. 5c-ii). Similarly, MRE11 also showed a marked reduction in expression levels following ROCK2 antibody treatment (Fig. 5d-i and 5d-ii). Recent literature has shown that actin is involved in DNA repair processes [63–65]. Since, canonically ROCK2 is involved in actin modulation we also assessed the effect of inhibition of ROCK2 on nuclear actin. Incidentally, no changes in the levels of nuclear actin were observed although the cytoplasmic actin levels did show a reduction, following inhibition of ROCK2. Notably, the pH2Ax levels diminished upon ROCK2 inhibition as expected (S5c(i-ii)). These observations convincingly implicate that ROCK2 regulates the early DNA repair assembly, confirmed by both the biochemical studies and transcriptomic analysis, however the exact mechanistic is yet to be understood.

### ROCK2 expression results in an increased cell survival and cell cycle transition

**Fig. 6:**
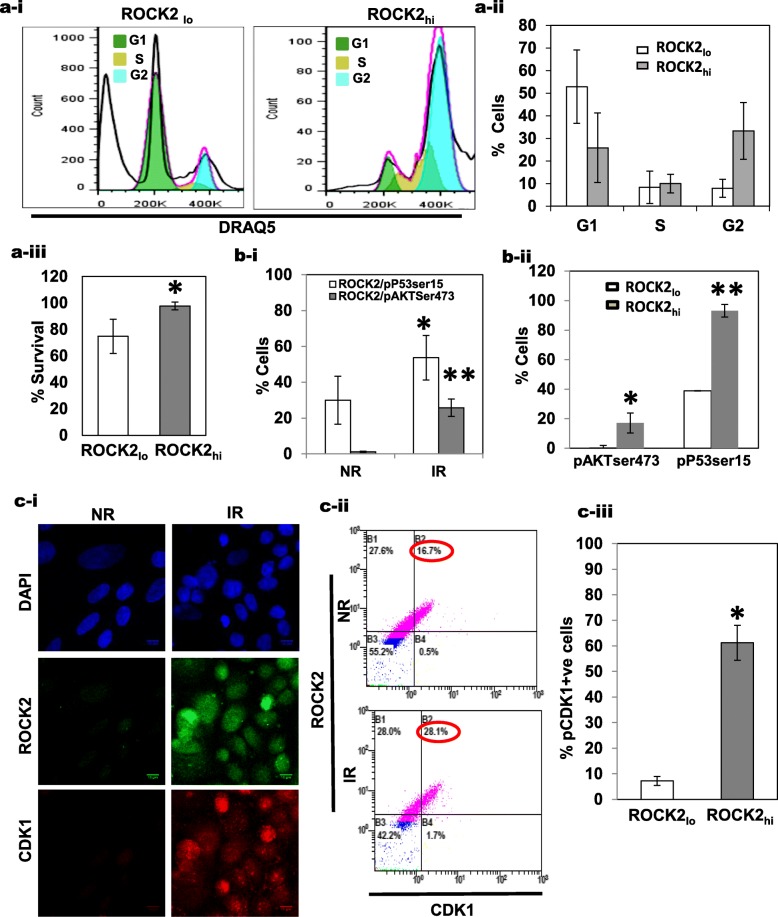
ROCK2_hi_ cells have an enhanced cell survival and are primed for a G2-M transition. **a-i** Representative histograms of flow cytometry analysis displaying the cell cycle profile of irradiated SiHa cells. The cell cycle plots were obtained after gating based on ROCK2 expression i.e., ROCK2 high expressing (ROCK2_hi_) cells and ROCK2 low expressing (ROCK2_lo_) cells. **a-ii** Graphical representation of distribution of cells in various phases of cell cycle, showing that the ROCK2_hi_ cells have a significant percentage of cells in G2/M phase as compared to the ROCK2_lo_ cells (*n* = 3). **a-iii** Graphical representation of percentage survival of ROCK2_hi_ cells and ROCK2_lo_ cells. ROCK2_hi_ cells shows a better survival (*n* = 3, * *p* < 0.02). **b-i** Graphical representation of the increased percentages of pAKT-Ser473 and P53-Ser15 in the SiHa cells following radiation treatment as compared to the untreated controls, *p < 0.03; **p < 0.03 (*n* = 3). **b-ii** Graphical representation of the distribution of pAKT-Ser473 and P53-Ser15 in ROCK2_hi_ population as compared to ROCK2_lo_ cells, gated based on ROCK2 expression levels in the irradiated SiHa cells, *p < 0.009; **p < 0.03 (*n* = 3). **c-i** Immunofluorescence staining showed an upregulated expression of both ROCK2 and CDK1 upon irradiation (scale bar = 10 μm). **c-ii** Flow cytometric analysis of irradiated SiHa cells showed an increased percentage of cells that co-expressed ROCK2 and CDK1 as compared to the corresponding control. **c-iii** Graphical representation of the distribution of pCDK1 levels in the ROCK2_hi_ population as compared to ROCK2_lo_, gated based on ROCK2 expression levels in the irradiated SiHa cells, *p < 0.003 (*n* = 3)

The transcriptomic data also shows that ROCK2_hi_ cells have better survival and cell cycle machinery. The GO annotation showed an enrichment of genes for the p53 mediated DNA damage response (Fig. 4c) indicating that these cells may have a better cell survival as also shown in Fig. 4a. Further studies were thus directed towards this investigation. Cell cycle profiles of the irradiated and non-irradiated SiHa cells showed a distinct G2/M peak as compared to the control cells (Additional file 1: Figure S6a). A flow cytometric analysis was performed on the cells stained for ROCK2 and a DNA-binding dye DRAQ5 to evaluate the cell cycle profiles. This revealed a distinct sub-G1 peak associated with the ROCK2_lo_ cells, indicating cell death in these cells, while the absence of a sub-G1 peak and the presence of a prominent G2/M peak was observed in the ROCK2_hi_ cells (Fig. 6a (i-iii)), implying that the ROCK2_hi_ cells have a selective survival advantage over the ROCK2_lo_ cells. To test this further, the expression levels of phosphorylated P53 (pP53-Ser15) and pAKT (pAKT-Ser473) were measured in irradiated and non-irradiated cells. pP53-Ser15 is shown to elicit DNA damage response when triggered by a DNA damage stimulus after the onset of the initial HR and NHEJ repair events and supports cell survival [66]. This phosphorylated form of p53 is also shown to be triggered by DNA damage stimulus and regulates the repair process by crosstalk with pH2Ax [67]. pAKT-Ser473 on the other hand is known to drive the downstream survival pathways endowing a survival advantage to the cells [68]. We observed that the percentage of cells positive for both ROCK2 and pAKT-Ser473 (ROCK2 + ve/pAKT-Ser473 + ve) or ROCK2 and pP53-Ser15 (ROCK2 + ve/pP53-Ser15 + ve), increased significantly upon irradiation (Fig. 6b-i). Further, ROCK2_hi_ cells were seen to have higher expression of pP53-Ser15 and pAKT-Ser473 as compared to ROCK2_lo_ cells under both irradiated and non-irradiated conditions (Fig. 6b-ii and Additional file 1: Figure S6b(i-ii). The above data supports the observation that ROCK2_hi_ cells have a superior survival mechanism as compared to ROCK2_lo_cells**.**

To test if the ROCK2_hi_ cells have an advantage in cell cycle progression and transition from G2 to M post-irradiation induced arrest, we investigated the expression of CDK1 in both populations. As shown in Fig. 4c, the transcriptomic data indicates that the genes facilitating G2/M transition are overexpressed in ROCK2_hi_ cells. CDK1 is an indicator of the progression of cells from the G2 to the M phase of the cell cycle [69–72]. Christopher Marshall’s group earlier reported that ROCK and CDK1 co-operate in tumor progression in melanoma and non-small cell lung cancer, in mice models [73]. Consistently, we found that the cell population, co-expressing ROCK2 and CDK1, increased post irradiation as observed by both immunofluorescence and flow cytometry (Fig. 6c(i-ii)). Significantly the ROCK2_hi_ cells had higher expression of phospho-Thr161 CDK1 (pCDK1) [74] as compared to ROCK2_lo_ cells, under both irradiated and non-irradiated conditions as revealed by flow cytometry (Fig. 6c-iii and Additional file 1: Figure S6b-iii). The existence of a pool of cells with high ROCK2 and pCDK1 reveals the enhanced cell cycling capability of these cells.

Reports from Wang et al., suggest a functional interaction between ROCK2 and BRCA2 in late events of the cell cycle, particularly during centromere duplication [75]. Such association was also observed in our study wherein BRCA2 was shown to co-express with ROCK2 in irradiated cells. The ROCK2 and BRCA2 proteins were found to have an increased interaction when subjected to irradiation (Additional file 1: Figure S6c and S6d). Our data thus affirms a role for ROCK2 in radiation response supported by better DNA repair, cell survival and cell cycle machinery.

### ROCK2 is the downstream effector of RhoC in the context of radiation response

**Fig. 7:**
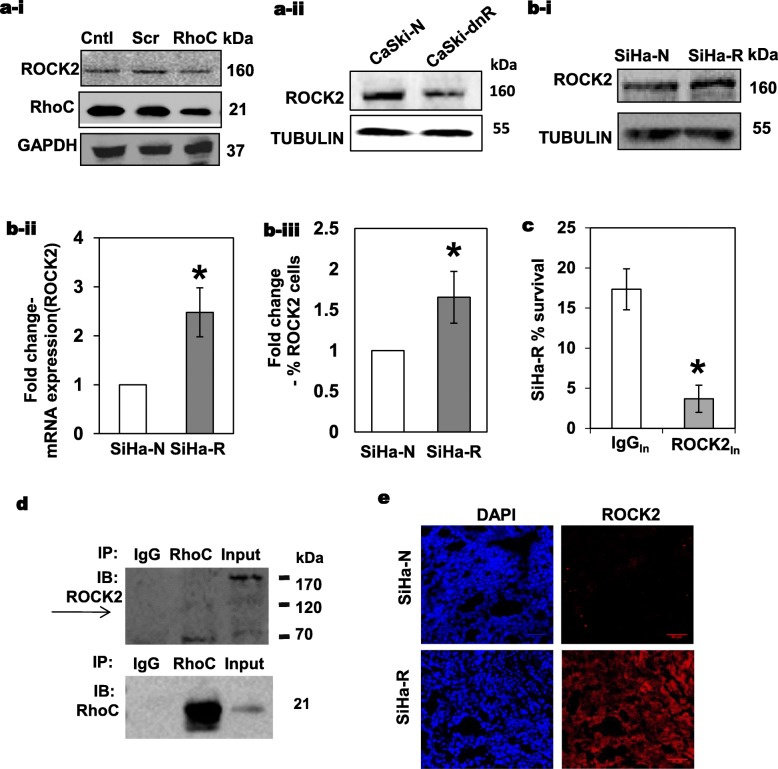
ROCK2 is the downstream effector of RhoC in radiation response. **a-i** An siRNA-based inhibition of RhoC in SiHa cells followed by immunoblotting of the cell extracts showed a reduction in the protein levels of RhoC and ROCK2 (*n* = 3). **a-ii** Immunoblotting the cell extracts of CaSki cells expressing the dominant negative RhoC (CaSki-dnR) showed a reduction in the ROCK2 levels as compared to the cells containing the empty vector (CaSki-N) (*n* = 3). **b-i** Immunoblotting of the cell extracts from SiHa cells with overexpression of RhoC showed an increase in the levels of ROCK2 as compared to the control cells (*n* = 3). **b-ii** Real time PCR showed a 2.4- fold upregulation of ROCK2 mRNA levels in SiHa-R cells as compared to SiHa-N cells (*n* = 3, *p* < 0.05). **b-iii** Graphical representation of flow cytometric analysis showed a 1.65-fold increase in the percentage of ROCK2 positive cells in the SiHa-R as compared to SiHa-N cells *p* < 0.05 (*n* = 3). **c** Graphical representation of flow cytometry analysis of SiHa-R cells with ROCK2_In_ displayed loss of survival advantage post-irradiation as compared to IgG_In_ cells (*n* = 3, *p < 0.03). **d** Immunoprecipitation using RhoC antibody resulted in pulldown of ROCK2 (~ 120 kDa). **e** Representative images of immunofluorescence-based analysis of xenografts derived from SiHa-N and SiHa-R tumors showing increased expression of ROCK2 in SiHa-R derived sections (scale bar = 50 μm)

The phenotypic similarity between RhoC and ROCK2 in terms of response to radiation is evident from the above data and suggests that ROCK2 may be an effector of RhoC in this context. As expected, RhoC knockdown using siRNA resulted in a subsequent decrease in ROCK2 expression in SiHa cells (Fig. 7a-i). We also observed that CaSki-dnR cells had lower ROCK2 expression as compared to CaSki-N cells (Fig. 7a-ii), suggesting that RhoC regulates the expression of ROCK2 in cervical cancer cells. Also, as expected, immunoblot analysis revealed that ROCK2 is upregulated in SiHa-R cells as compared to SiHa-N cells (Fig. 7b-i), which was also confirmed by a qPCR analysis (Fig. 7b-ii). Alternatively, flow cytometric analysis of SiHa-R and SiHa-N cells for ROCK2 showed that percentage of cells with ROCK2 expression were higher in SiHa-R cells (Fig. 7b-iii). It is well established that RhoC is a regulator of ROCK2 functionally, however, this is the first report which suggests that the expression of ROCK2 is altered by RhoC, though the mechanism remains unknown and is under investigation. In order to confirm that ROCK2 is the downstream effector of RhoC in radioprotection, we inhibited ROCK2 function using antibodies in SiHa-R cells, which have been proven to have a strong radioresistant phenotype. As expected, the inhibition of ROCK2 resulted in abrogation of the radioprotective effect mediated by RhoC as shown in Fig. 7c. This rescue of phenotype study confirmed that ROCK2 is indeed the downstream target of RhoC in the context of radiation response. We further investigated if ROCK2 and RhoC physically interact with each other in the nuclear compartment of the cervical cancer cells. As evident from Fig. 7d, there was co-immunoprecipitation of ROCK2 protein when RhoC was pulled down from the nuclear fraction. We do not see a prominent 160 kDa pull down, however a 120 kDa band is observed in the RhoC pull down lane as compared to the isotype IgG pull down. Xenograft sections of SiHa-R and SiHa-N were also assayed to analyze the expression changes of ROCK2, in vivo, following alterations in RhoC expression. We observed that SiHa-R sections had higher ROCK2 expression as compared to sections obtained from SiHa-N xenografts (Fig. 7e).

**Fig. 8:**
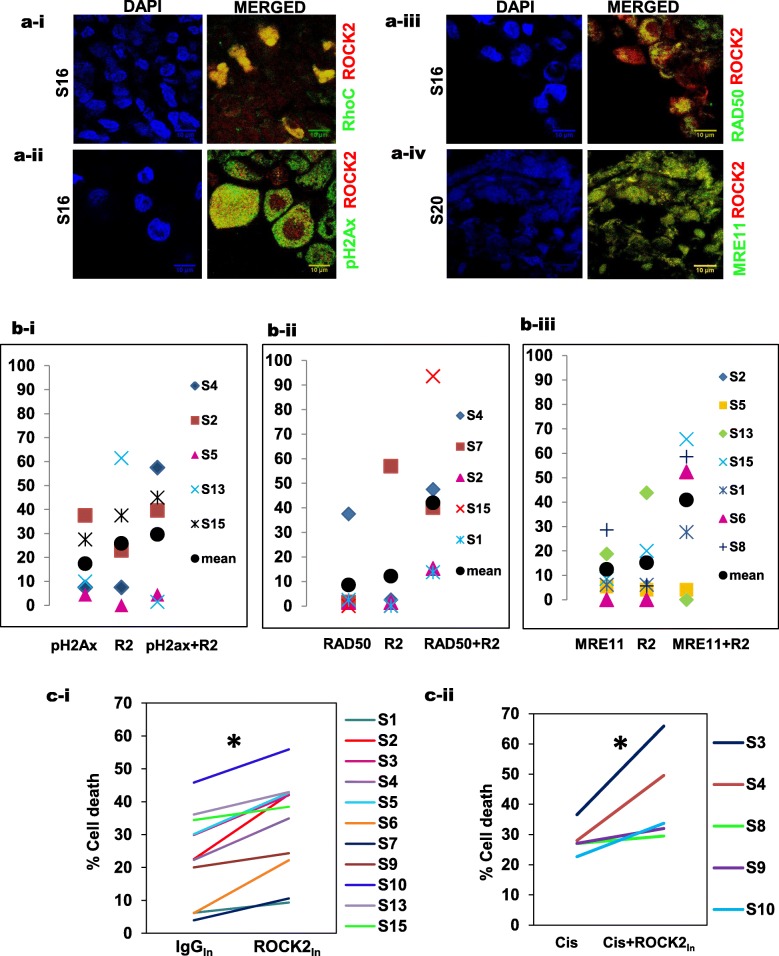
Evaluation of ROCK2 as a prospective radiosensitizer in vitro using clinical samples and its association with DNA repair markers. **a-i** Immunofluorescence analysis shows co-expression of both RhoC and ROCK2 in the nuclear compartment of tumor sections (scale bar = 10 μm). **a-ii** Immunofluorescence analysis shows co-expression of both pH2Ax and ROCK2 in the nuclear compartment of tumor sections (scale bar = 10 μm). **a-iii** Immunofluorescence analysis shows co-expression of both RAD50 and ROCK2 in the nuclear compartment of tumor sections (scale bar = 10 μm). **a-iv** Immunofluorescence analysis shows co-expression of both MRE11 and ROCK2 in the nuclear compartment of tumor sections (scale bar = 10 μm). **b(i-iii)** Scatter plots depict the percentage of cells that showed nuclear expression of either ROCK2 or pH2Ax or both (R2 + pH2Ax). Similarly, cells were analyzed for nuclear expression of ROCK2-RAD50 and ROCK2-MRE11 as represented in the figure. **c-i** ROCK2_In_ was performed in cervical cancer biopsy derived tumor cells with a corresponding IgG_In_ control (*n* = 15 samples). Graphical representation of *n* = 11 samples with ROCK2_In_ resulted in increased cell death as compared to the control, is shown in the figure *p* < 0.0001. **c-ii** ROCK2_In_ performed in cervical cancer biopsy derived tumor cells along with Cisplatin (Cis) at 20 μM followed by irradiation showed an increased cell death. The graphical representation displays an increased cell death upon combinatorial treatment of cisplatin and ROCK2 as opposed to cisplatin alone (*n* = 5 samples)

In order to validate the clinical relevance of our study wherein ROCK2 in association with RhoC regulates radiation response of tumor cells via modulation of DNA repair proteins we performed studies on clinical samples. Assessment of the expression of RhoC and ROCK2 in tumor-derived sections showed that both RhoC and ROCK2 were coexpressed in a subpopulation of tumor cells. Interestingly, the co-expression was also found in the nuclear compartment of these cells as shown in Fig. 8a-i. It is important to note that RhoC, which is predominantly a cytosolic protein is observed in nuclear compartments of some of the tumor cells, an observation that has not been reported earlier. Similarly, the expression of ROCK2 and DNA repair proteins like pH2Ax (8a-ii), RAD50 (8a-iii) and MRE11 (8a-iv) was also analyzed in the clinical sections. The analysis of the stained sections revealed the existence of tumor cells that showed expression of either ROCK2 or DNA repair markers or both in the nuclear compartment. However, the percentage of cells that co-expressed ROCK2 and either of the DNA repair markers (MRE11, RAD50 and pH2Ax) in the nuclear compartment was higher as depicted in Figs. 8b(i-iii), indicating a potential interaction of these proteins.

To further explore if ROCK2 could be a potential radiosensitization target, clinical samples were inhibited for ROCK2 and their response to radiation was analyzed, in vitro. The clinical specimens were disintegrated into a single cell suspension which was followed by a ROCK2 inhibition, using the antibody-based technique described earlier. The results suggest a significant increase in cell death over the IgG treated control cells (Fig. 8c-i). Concomitantly; cells were also treated with Cisplatin (20 μM) as concurrent chemo-radiation is the current treatment modality for cervical cancer. It was observed that additive treatment of cells with Cisplatin and ROCK2 inhibition resulted in a significant increase in cell death upon irradiation as compared to cells with Cisplatin treatment alone (Fig. 8c-ii).

Thus, our study establishes that RhoC regulates radio-resistance in cervical cancer via ROCK2’s crosstalk with DNA repair assembly proteins, however the exact mechanism of regulation of ROCK2 by RhoC is still under investigation.

## Discussion

Existing literature supports the hypothesis that heterogeneous response of tumor cells to radiation is due to existence of a subpopulation of cancer cells which are resistant to radiation [76]. The combination of intrinsic properties including DNA repair, cell cycle status, survival pathways and extrinsic properties like stimulus from the microenvironment enable these cells to withstand radiation assault. There is surmounting data suggesting that a cell’s increased genomic stability is attained by utilizing DNA repair machinery. Understanding these processes and their mechanisms contribute to development of effective therapeutic strategies targeting advanced cancers. Although it is well accepted that RhoA plays an important role in radiation response [77], the role of RhoC, a RhoGTPase family protein, has not been explored in this context. RhoC and its downstream effector ROCK2 have been independently shown to regulate tumor progression in several tumor types [22, 24, 35, 49, 78–80]. Although ROCK2 plays an important role in cell division, the precise role of ROCK2 in DNA repair remains elusive. Our data proposes a novel role of RhoC in cervical cancer radioresistance and presents evidence to support ROCK2 as the downstream effector of RhoC in radioresistance.

Our key finding is that enhanced RhoC levels contribute to radioprotection in cervical cancer cells. It modifies the transcriptional network associated with DNA repair and cell cycle progression. ROCK2, the downstream effector of RhoC, crosstalks with DNA repair machinery to regulate radiation response, and its inhibition in primary tumors results in radiosensitization, in vitro. We also report that both RhoC and ROCK2 are localized in the nuclear compartment in a subset of tumor cells.

A recent report implicates RhoC in chemoresistance in non-small cell lung cancer [25], however its role in radioresistance is not elucidated in any tumor model. We have earlier reported that RhoC regulates several tumor phenotypes, and in this study, we extend the role of RhoC to radioresistance mediated through transcriptional regulation of DNA repair machinery.

Following irradiation, the DNA damage checkpoint responses and DNA repair machinery are important in cellular response to radiation [59, 81]. We show that RhoC contributes to radiation response in tumor cells by regulating the expression of important genes involved in DNA repair machinery. The comparison of DNA repair genes in SiHa cells with RhoC overexpression showed a marked upregulation of important DNA repair proteins including RAD50 and MRE11, essential components of the MRN complex [62]. The overexpression of RhoC also led to increased levels of pH2Ax in SiHa cells while its inhibition resulted in reduced pH2Ax foci formation. The pH2Ax foci formation is the hallmark for radiation-induced DSBs in DNA and is also used to understand DNA repair kinetics [82]. Our in vitro observations were supported by a transcriptomic analysis of the SiHa cells with stable overexpression of RhoC, which also revealed enrichment of genes regulating the DNA repair pathway. It is important to note that elevated RhoC expression resulted in a gamut of changes in various biological processes including cell proliferation, chromatin remodeling, planar cell polarity and cell cycle arrest, but importantly several biological processes related to DNA repair were also highlighted. G1/S transition, DSB repair via NHEJ pathway and cellular response to DNA damage were biological processes that were enriched in SiHa-R cells. Irrespective of detailed mechanistic, the findings herein demonstrate that RhoC selectively upregulates the transcriptional network of DNA repair.

Amongst the various downstream effectors of Rho GTPases, the Rho associated coiled coil kinases (ROCKs) play an important role in cancer progression and ROCK2 has been shown to be present in the nuclear compartment and is involved in the cell division process [34, 83, 84]. Further experiments were thus directed to discern the role of ROCK2 as the downstream effector of RhoC in cervical cancer radioresistance. A major consequence of inhibition of ROCK2 was increased radio-sensitization and consequently increased cell death of cancer cells, in vitro. Additionally, increased expression of ROCK2 enhanced cell survival post-irradiation. Importantly, the inhibition of ROCK2 resulted in abolition of radioresistant phenotype gained by SiHa-R cells. The finding that ROCK2 and RhoC have phenotypically similar consequences on radioresistance suggested that this pathway regulates DNA damage response induced by ionizing radiation. Intriguingly, a transcriptomic profiling of the ROCK2_hi_ cells showed similar biological process enrichment as that observed in SiHa-R cells.

There are several reports on the role of ROCK2 in tumor progression and in normal differentiation [85–88]. However, its role in radioresistance in the context of cervical cancer reported herein is a novel finding. It has been reported in hepatocellular carcinoma that ROCK2 regulates the cell division process by regulation of the ubiquitination of Cdc25A, a G1/S transition protein [49]. The most interesting observation was reported by Wang et al., wherein they showed that BRCA2 and nucleophosmin (NPM) regulate centrosome duplication by forming a complex with ROCK2 [84]. Also, ROCK2 has been shown to regulate centrosome duplication by modulation of CDK2/CyclinE complex [35]. Though its interaction with the cell cycle regulatory machinery is well explored, the cross talk with DNA repair proteins has not been reported till date.

Radiation induced repair of damaged DNA by activation of DNA damage checkpoints, results in cell cycle arrest to enable the DNA repair process. The initial events comprise of sensing the damage followed by activation of p53 which results in cell cycle arrest at G1/S and G2/M allowing the cells to repair the DNA. A number of proteins like ATM, BRCA2/1, CHEK1/2 and RAD family proteins are involved in these processes [59]. Additionally, pH2Ax foci formation is the hallmark DSB marker and also an early sensor of DNA damage [45]. Our study extends the role of ROCK2 to DNA repair by presenting evidence of its crosstalk with the early DNA repair assembly proteins including pH2AX and the MRN complex. Inhibition of ROCK2 resulted in reduced pH2AX foci formation and MRE11-RAD50 proteins of the MRN complex which was also supported by transcriptomic analysis of the ROCK2_hi_ and ROCK2_lo_ cells, which displayed an upregulation of DNA repair proteins and enrichment of the DNA repair related biological processes. Our data clearly indicates that ROCK2 intercalates with the early DNA damage response machinery. However, irrespective of the mechanism of interaction with the early repair machinery, these findings support the role of ROCK2 in the early DNA repair process. It is intriguing to note that ROCK2 cooperates with several nuclear proteins to participate in DNA homeostasis related processes and is thus a promising target for biomarker development.

The consequences of ROCK2 inhibition was also validated using clinical samples wherein we found an increased radiosensitization of tumor cells. But the observation that ROCK2 inhibition and Cisplatin together radiosensitized the tumor cells better than Cisplatin alone, was the most exciting revelation. It enables us to take this study further and check the potential of the use of ROCK2 inhibition and Cisplatin, in vivo in mice models, and on a larger cohort to establish it as a therapeutic target.

The observation which was equally important to note was the expression of RhoC in nuclear compartments of the tumor cells. RhoC is classically a cytosolic protein which regulates cytoskeletal organization [89] and cancer cell motility [90]. There is only one report which indicates the presence of RhoC in nuclear compartment in breast cancer cell lines [19]. The observation that a fraction of RhoA is found in the nucleus and is activated by Net1 upon irradiation [91] lends support to our finding that RhoC, which is homologous to RhoA, may exist in the nucleus. Irrespective of the lack of exact mechanism of activation of nuclear RhoC, it is important to note that a subset of tumor cells, in the clinical samples, exhibit strong co-expression of ROCK2 and RhoC. The presence of RhoC is important in this context, wherein it might be required for activation of ROCK2 in the nucleus. The co-expression of the two proteins intuitively points towards the existence of a molecularly distinct tumor cell population. Similarly, the cells that are dual positive for ROCK2 and DNA repair may be identified as cells with better DNA repair abilities which may have intrinsic resistance ability, consequently leading to therapy evasion and relapse. Such cells with enhanced radioresistance are believed to be cancer stem cells which exhibit heterogeneity in their radiation response [92–96]. The co-immunoprecipitation of ROCK2, from nuclear fraction of the cells, along with RhoC supports our finding. However, the co-precipitated ROCK2 is ~ 120 kDa as opposed to 160 kDa of ROCK2. Studies on Rho kinases (ROCK1 and ROCK2) [97, 98] have shown the presence of a smaller fragment of ROCK, ~ 130 kDa, in the cells under various cytological conditions. However, in the context of radioresistance the form of ROCK2 that binds to RhoC needs thorough investigation.

Finally, findings herein strongly provide robust evidence, both clinical and cell line based, that RhoC and ROCK2 regulate radiation response and contribute to radioresistance in cervical cancer. Presently, there is no specific biomarker to stratify cervical cancer tumor for therapy response. As compared to breast tumors where expression of various markers (ER, PR, and Her2) can be a determinant of the therapy outcome [99], cervical cancer does not have a robust marker. RhoC and ROCK2 have the potential to be developed as candidate biomarkers for radioresistant tumors to predict therapy outcome. Combined, our findings suggest a non-canonical signaling pathway for RhoC which regulates radiation response in cervical cancer via ROCK2 mediated DNA repair regulation. Future work would aim at evaluating ROCK2 as a biomarker and therapeutic target in the context of radioresistance in cervical cancer and understanding the mechanistic of RhoC and ROCK2 in DNA repair regulation.

## Conclusions

In this report, we investigated the role of RhoC and ROCK2 in radioresistance in cervical cancer. We have shown that the inhibition of RhoC and ROCK2 sensitizes tumor cells to irradiation. Based on transcriptomic analysis, we find that the gene network supporting DNA repair is enriched in RhoC overexpressing cells and this is also true for ROCK2_hi_ cells, which have an enrichment of genes supporting DNA repair, cell survival and cell division. ROCK2 operates by crosstalk with early DSB repair proteins. We also propose that inhibition of ROCK2 abrogates the gain of phenotype, radioresistance, which is conferred by overexpression of RhoC, thus placing it as the downstream effector of RhoC in this context. Most interestingly, the inhibition of ROCK2 along with Cisplatin induced a better sensitization to irradiation as compared to cisplatin alone, in a fraction of clinical samples. Thus, the two molecules have the potential to be developed as biomarkers or therapeutic targets for the enhancement of response to radiation therapy in this cancer.

## Additional file

Additional file 1: Supplementary figures. (ZIP 9690 kb)

## Data Availability

Both raw and processed data analyzed in this study is available upon request.

## Abbreviations

CCRT: Concurrent chemoradiation
DAPI: 4,6-diamidino-2-phenylindole
DMEM: Dulbecco’s Modified Eagle Medium
EDTA: Ethylenediaminetetraacetic acid
FBS: Fetal Bovine Serum
LINAC: Linear accelerator
Neo: Neomycin
NPM: Nucleophosmin
pCDK1: CDK1pThr161
PFA: Paraformaldehyde
pH2Ax: pSer139-H2Ax
PI: Propidium Iodide
ROCK: Rho-associated kinases
ROCK2_hi_: ROCK2 high cells
ROCK2_lo_: ROCK2 low cells
SCC: Squamous cell carcinoma
SiHa-N: SiHa Neo cells
SiHa-R: SiHa RhoC cells
