# Supplementary material for: RhoC regulates radioresistance via crosstalk of ROCK2 with the DNA repair machinery in cervical cancer
Source: J Exp Clin Cancer Res. 2019 Sep 5;38:392. doi: 10.1186/s13046-019-1385-7 (PMC6729006; doi:10.1186/s13046-019-1385-7)
Supplement: Supplementary file 1 — Supplementary figures. (ZIP 9690 kb) [file 13046_2019_1385_MOESM1_ESM.zip › 13046_2019_1385_MOESM1_ESM/Supplementary Figure Legends_revised.docx]

**Supplementary Figure Legends:**

**Supplementary Figure 1**

**(a)** Graphical representation of flow cytometry analysis of CaSki-dnR cells showing a higher sensitization to irradiation as compared to CaSki-N control cells using propidium iodide (PI). A 0.61-fold reduction was detected in the survival of CaSki-dnR cells *, p<0.01 (n=3).

**(b-i) A** WST (cell viability test) analysis to arrive at the dose response curve for SiHa cells under various doses of radiation over 7 days is represented.

**(b-ii)** Graphical representation of the fold ratio of survival at 6Gy, which was chosen as the LD50 value (n=3, p<0.05).

**(c)** Immunofluorescence analysis of the pH2Ax protein post-irradiation indicating foci formation in the IR cells. scale bar=10μm. (n=3).

**d(i-ii)** Survival analysis by using Annexin V and PI followed by flow cytometry confirmed radioresistance of re-irradiated cells. The quadrant plots depicted the live population individually in NR (non-irradiated), IR (irradiated) and re-IR (re-irradiated cells) are shown in the figure (n=3).

**(e-i)** Soft agar colony formation assay has shown a 15-fold increase in number of larger colonies in the IR cells (n=3, p<0.01).

**(e-ii)** Migration assay indicated that IR cells have better migration potential as compared to NR cells (n=3, p<0.05).

**Supplementary Figure 2**

**(a-i)** Real-time PCR analysis showed a 1.6-fold up-regulation in the mRNA expression of ROCK2 in IR as compared to NR cells p<0.05, (n=3).

**(a-ii)** Immunofluorescence assay showed that there is an up-regulation of RhoC in the IR SiHa cells as compared to the NR cells (scale bar=25μm).

**(b-i)** Representative image of immunofluorescence analysis of RhoC in RhoC siRNA treated (50picomoles) and control (scrambled siRNA) cells (scale bar=25μm).

**(b-ii)** Immunoblot analysis of Scrambled siRNA and RhoC siRNA transfected cell extracts showed reduction in RhoC protein levels (n=3).

**(c)** Immunofluorescence analysis of DNA repair protein, pH2Ax showed an increased expression level in SiHa-R as compared to SiHa-N xenograft derived tumor sections (scale bar=50 μm).

**(d)** Cellular extracts of CaSki-N and CaSki-dnR were assessed for RAD50 protein expression using western blotting. RAD50 showed a reduced expression in CaSki-dnR cells (n=3).

**Supplementary Figure 3**

**(a)** Immunoblot of SiHa IR and Re-IR cell extracts showed an increased expression of ROCK2 and RhoC proteins.

**(b)** Densitometric analysis of nuclear fraction blots shown in Figure 3c confirmed the increased nuclear levels of ROCK2 protein on D1(24hour) and D2(48hour) as compared to the 0hour control cells (n=4)

**(c)** Pre-extraction followed by immunofluorescence for ROCK2 showed its localization to the nuclear compartment as compared to the NR cells. The nuclei are marked using Image J software (scale bar=10 μm).

**(d)** Overexpression of pCAG-ROCK2 plasmid was confirmed using a qPCR analysis. pCAG empty vector was used as the control.

**(e-i)** Flow cytometric analysis using Annexin V and PI staining showed an increase in Annexin V positive cells (early apoptosis) upon siRNA- based inhibition of ROCK2 followed by irradiation (n=3, *p<0.05). Scr- Scrambled siRNA; ROCK2- ROCK2 siRNA

**(e-ii)** Immunoblotting analysis confirmed reduction in ROCK2 levels upon siRNA knockdown (n=3).

**(f)** Flow cytometric analysis of using Annexin and PI staining showed that saponin treatment did not alter the viability of the SiHa cells upon irradiation (n=3).

**(g)** Immunoprecipitation using dynabeads showed that the ROCK2 antibody specifically bound to the intracellular ROCK2 antigen.

**(h-i)** Representation of mice xenograft tumors formed using NR and IR cells that were injected subcutaneously into SCID mice. Tumor formation was allowed for 4 weeks.

**(h-ii)** Immunoblot of cell extracts from NR and IR xenografts showed enhanced ROCK2 levels in the IR tumor derived cells.

**Supplementary Figure 4**

**(a)** Representative flow cytometry histogram plot showing the distribution of cells stained for ROCK2 and gated for ROCK2_lo_ and ROCK2_hi_ populations. Secondary control was used to set the gates. Gate C- ROCK2_lo;_ Gate D- ROCK2_hi_

**(b)** Representative images of immunofluorescence analysis confirming that the live sorted ROCK2_hi_ and ROCK2_lo_ cells indeed have higher and lower expressions of ROCK2 respectively (scale bar=10μm).

**(c-i)** qPCR analysis to validate the ROCK2 levels in the live sorted ROCK2_hi_ and ROCK2_lo_ cells (p<0.05; n=3).

**(c-ii)** qPCR analysis for RhoC expression levels in the live sorted ROCK2_hi_ and ROCK2_lo_ cells showed an up-regulation of RhoC in the ROCK2_hi_ sorted population (p<0.05; n=3).

**(d)** Heatmap representation of differentially expressed genes in the ROCK2_hi_ and ROCK2_lo_ cells using Clustvis, R-based webtool.

**Supplementary Figure 5**

**(a)** Representative images of immunofluorescence analysis of NBS1, MRE11, RAD50, DNA-PK and ATM in IR and NR SiHa cells (scale bar=25 μm)

**(b)** Immunoprecipitation using ROCK2 antibody showed the interaction with pH2Ax following irradiation treatment.

**(c-i)** Immunoblot representation of the reduction in the nuclear pH2Ax in the ROCK2_In_ cells as compared to the vehicle control. The Actin levels did not show any reduction in the nuclear fractions. Tubulin and H3 served as controls for cytoplasmic and nuclear fractions respectively.

**(c-ii)** Immunoblot depicts the reduction in the actin levels following ROCK2 inhibition in the cytoplasmic fractions. Tubulin and H3 served as controls for cytoplasmic and nuclear fractions respectively.

**Supplementary Figure 6**

**(a)** Representation of cell cycle profiles of NR and IR cells using DRAQ5, a DNA binding dye.

**b(i-iii)** Overlay plots depicting the increased percentage distribution of pAKT, pP53 and pCDK1 in the ROCK2 _hi_ cells as compared to the ROCK2_lo_ cells in the non-irradiated cells.

**(c)** Increased expression of ROCK2 and BRCA2 in the IR cells as compared to the NR cells. scale bar=10μm. (n=3)

**(d)** Immunoprecipitation using ROCK2 antibody and immunoblotting with BRCA2 antibody confirms the enhanced interaction of these proteins following irradiation
